# Morphine Reward Promotes Cue-Sensitive Learning: Implication of Dorsal Striatal CREB Activity

**DOI:** 10.3389/fpsyt.2017.00087

**Published:** 2017-05-30

**Authors:** Mathieu Baudonnat, Jean-Louis Guillou, Marianne Husson, Veronique D. Bohbot, Lars Schwabe, Vincent David

**Affiliations:** ^1^CNRS UMR 5287, Institut de Neurosciences Cognitives et Intégratives d’Aquitaine, Pessac, France; ^2^Département des Sciences de la Vie et de la Santé, Nouvelle Université de Bordeaux, Pessac, France; ^3^Department of Psychiatry, Douglas Institute, McGill University, Montreal, QC, Canada; ^4^Department of Cognitive Psychology, University of Hamburg, Hamburg, Germany

**Keywords:** reward, drug self-administration, CREB, memory, morphine, striatum, ventral tegmental area

## Abstract

Different parallel neural circuits interact and may even compete to process and store information: whereas stimulus–response (S–R) learning critically depends on the dorsal striatum (DS), spatial memory relies on the hippocampus (HPC). Strikingly, despite its potential importance for our understanding of addictive behaviors, the impact of drug rewards on memory systems dynamics has not been extensively studied. Here, we assessed long-term effects of drug- vs food reinforcement on the subsequent use of S–R vs spatial learning strategies and their neural substrates. Mice were trained in a Y-maze cue-guided task, during which either food or morphine injections into the ventral tegmental area (VTA) were used as rewards. Although drug- and food-reinforced mice learned the Y-maze task equally well, drug-reinforced mice exhibited a preferential use of an S–R learning strategy when tested in a water-maze competition task designed to dissociate cue-based and spatial learning. This cognitive bias was associated with a persistent increase in the phosphorylated form of cAMP response element-binding protein phosphorylation (pCREB) within the DS, and a decrease of pCREB expression in the HPC. Pharmacological inhibition of striatal PKA pathway in drug-rewarded mice limited the morphine-induced increase in levels of pCREB in DS and restored a balanced use of spatial vs cue-based learning. Our findings suggest that drug (opiate) reward biases the engagement of separate memory systems toward a predominant use of the cue-dependent system *via* an increase in learning-related striatal pCREB activity. Persistent functional imbalance between striatal and hippocampal activity could contribute to the persistence of addictive behaviors, or counteract the efficiency of pharmacological or psychotherapeutic treatments.

## Introduction

Drug addiction may be viewed as an aberrant form of learning during which strong associations linking actions to drug seeking are expressed as persistent stimulus–response (S–R) habits, thereby increasing the vulnerability to relapse (1–3). Whereas the hippocampal memory system encodes relationships between events and their later flexible use, the dorsal part of the striatum plays a critical role in habit/procedural learning (4–7). Studies in both rodents and humans support the view that the hippocampus (HPC) and the dorsal striatum (DS) interact in either a cooperative (8–10) or competitive manner during learning (11–14). It is well documented that emotional, stressful events are potent modulators of striatum–HPC interactions: they promote habitual over cognitive forms of learning, through the interaction of glucocorticoids and noradrenaline (15–19). The amygdala plays a key role in orchestrating the switch from hippocampal to striatal learning (20, 21). Stress decreases hippocampal LTP in rodents with an intact amygdala, but not in lesioned animals (22). In contrast, we know surprisingly little about the impact of rewards on interactions between memory systems.

All rewards, whether they are sensory (e.g., food) or pharmacological (e.g., drugs of abuse), activate an ascending dopamine (DA) mesolimbic circuit composed of neurons projecting from the ventral tegmental area (VTA) to the nucleus accumbens (NAC) (23, 24). This circuit mediates appetitive learning (25, 26) and is implicated in the transition from goal directed to habitual behavior through a succession of loops recruiting progressively the nigrostriatal system following novelty-elicited activation of the mesolimbic pathway (27–30). The VTA also provides direct innervation to the HPC forming a loop that could act as a gating mechanism allowing access to long-term memory (31, 32). The VTA therefore appears to be a key locus for modulating interactions between memory systems (33, 34). We have previously reported that drug, but not food rewards lead to a deficit in a spatial memory task, while sparing a cued version of the same task (35). These effects were related to an increase in the PKA dependent phosphorylation of the cAMP response element-binding protein (pCREB) in the DS. pCREB is involved in the acquisition/consolidation of both cue-guided, striatum-dependent learning and spatial, HPC-dependent learning (12, 36–40). Interestingly, spatial learning produces transient waves of pCREB in the HPC, and a long-term increase in pCREB levels lasting up to 72 h (41). pCREB has been linked to synaptic plasticity changes and to late-long-term potentiation (l-LTP) (42, 43). The l-LTP is clearly involved in long-term memory formation (44), and DA is a potent modulator of these cellular adaptations (45, 46), further suggesting that the reward system modulates interactions between different forms of learning. These cellular adaptations may reinforce information processing by a particular memory system and thereby, determine the mode of learning strategies subsequently used.

In the present study, we investigated the impact of drug-induced activation of the reward system on the subsequent use of different learning strategies, i.e., HPC-dependent spatial vs striatum-dependent cue learning. We first tested the acquisition of a cued Y-maze discrimination task in animals rewarded with either food or intra-VTA drug self-injections. To compare the impact of these two forms of reward on subsequent learning processes, we then evaluated the preferential use of cued vs spatial learning strategies in a competition task and linked this preference to brain regional pCREB phosphorylation. We used two subsequent, different tasks to avoid direct drug-related effects on performance and to assess new learning as opposed to the expression of a consolidated memory. Finally, we tested whether pharmacological manipulation of the PKA/CREB pathway within the dorsal striatum (DS) can modulate learning strategies in animals with a history of drug self-administration.

## Animals and Methods

### Experiment I: Effects of Drug vs Food Reward on Learning Strategies

#### Animals

Male C57BL/6J mice (13 weeks old; Charles River) were housed individually and maintained on a 12 h light–dark artificial cycle (lights on at 7:00 a.m.) in a temperature-controlled colony room (22 ± 1°C). They were provided with food and water *ad libitum*. The week before behavioral testing, the food ration was adjusted individually so that animals reached 95% of their *ad libitum* weights during the Y-maze task. Immediately after the end of Y-maze testing, food was provided back *ad libitum*. All experiments were approved by the local Ethics Committee for Animal Experiments (Comité d’Ethique pour l’Expérimentation Animale de Bordeaux, CEE50) and were performed in accordance with the European Communities Council Directive of 1st February 2013 (2010/63/UE).

#### Surgery

**Figure 1:**
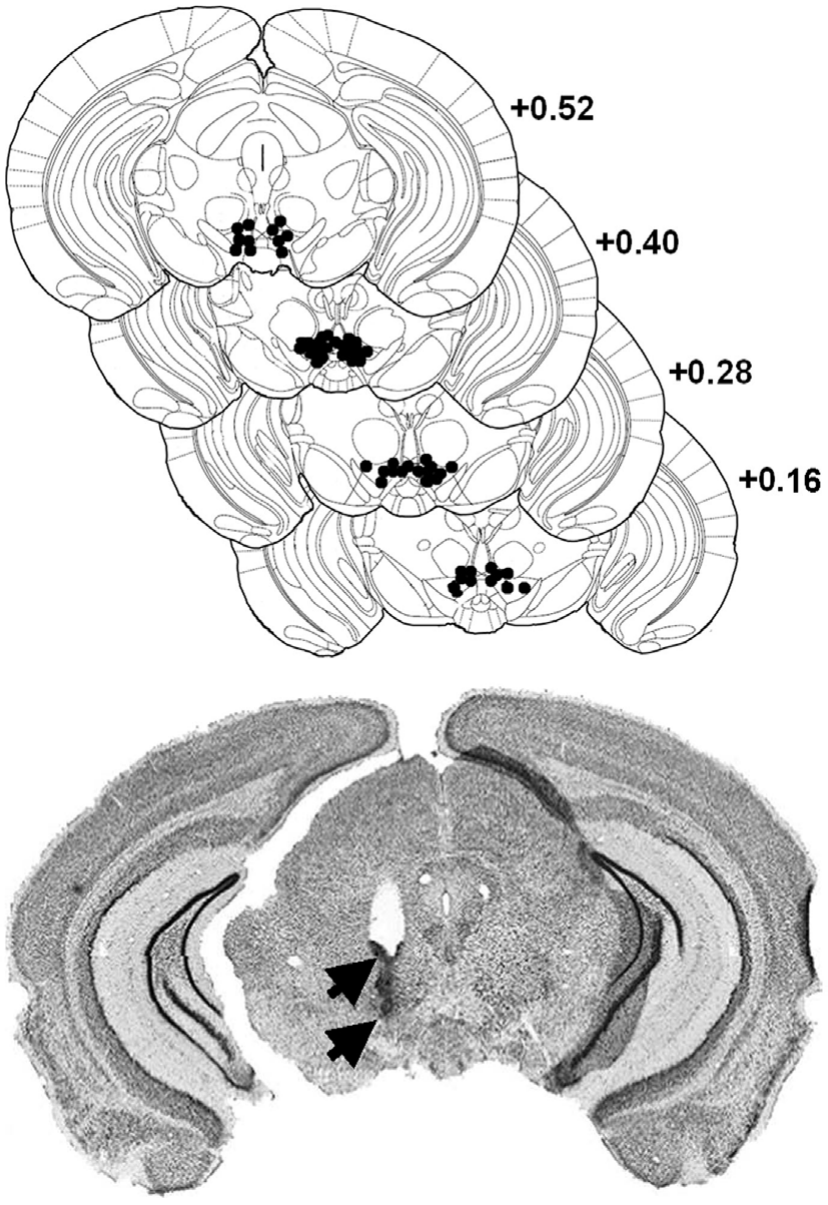
**Localization of morphine injection sites in the ventral tegmental area (VTA)**. *Top*: histological control of all implanted mice. Black dots show locations of the tip of the cannula (stereotaxic coordinates: AP, +0.4 mm from interaural; ML, ±0.3 mm; DV, +3.3 mm). Distribution of self-injection sites corresponds mainly to the posteromedial VTA projection system (23). *Bottom*: representative microphotograph of one injection site, showing traces left by the guide cannulae (upper arrow) and injection cannula (lower arrow).

Mice were anesthetized with a ketamine/xylazine mixture (Ketamine 1000 Virbac^®^: 100 mg/kg/Rompun^®^ 2%: 8 mg/kg i.p.), and lidocaine HCl (Xylocaine^®^, 5%) was applied locally before opening the scalp and trepanation. The incisor bar was leveled with the interaural line. A guide cannula (30 gauge, Le Guellec^®^, Douarnenez, France) is implanted unilaterally in a counterbalanced left and right order 1.5 mm above the posterior VTA (from interaural line: AP: +0.40 mm, ML: ±0.30 mm, DV: −3.30 mm from skull surface). Mice were allowed to recover from surgery for 1 week. After experiments, animals were anesthetized with Avertin (10 ml/kg, i.p.) and perfused transcardially with 4% paraformaldehyde in 0.1 M phosphate buffer (PB) for the histological control of all surgical implantations (see Figure 1) using thionin blue coloration (35).

#### The Y-Maze Task

**Figure 2:**
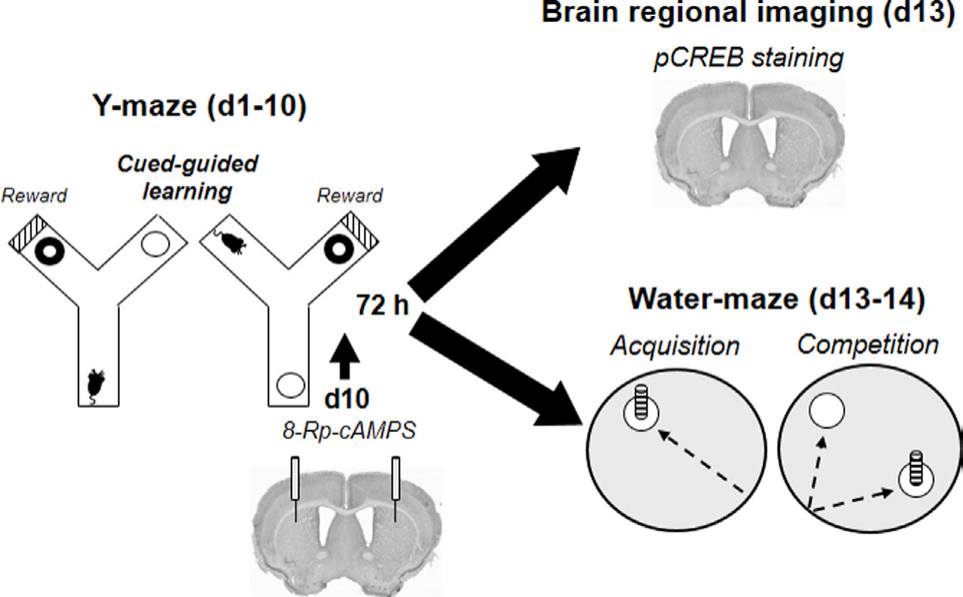
**Organization of behavioral, pharmacological, and CREB-imaging experiments**. Following habituation sessions (H1–H2), mice (*n* = 63) were trained for 10 days in a cued Y-maze discrimination task rewarded with either food or intra-ventral tegmental area morphine. Seventy-two hours after the last Y-maze training session, brains of the first cohort of animals (*n* = 30) were removed to proceed to pCREB immunostaining. A second cohort (*n* = 33) was trained in the water-maze competition task 72 h after completion of the Y-maze training. During the acquisition phase (d13), animals had to retrieve a submerged platform located by a cue from a constant starting point. The day after (d14), they were submitted to the competition test during which each mouse started their trials from a new location and have to choose between a spatially located and a cued platform. The third cohort received the PKA inhibitor Rp-8Br-cAMPS bilaterally into the dorsal striatum immediately before the last Y-maze training session (d10). They were then trained in the water-maze competition task (d13–d14) 72 h after completion of Y-maze acquisition.

All procedures started with a 10-day Y-maze training protocol and are schematized in Figure 2. The Y-maze discrimination protocol was identical to the one described in Ref. (35). Briefly, animals (*n* = 47) had to learn that a visual intra-maze cue (black–white striped laminated paper) is associated with the delivery of reward. They were separated into four groups: the first group was rewarded using a self-administration system allowing the delivery of microinjections of morphine into the VTA (morphine reward: 50 ng/50 nl/inj, *n* = 17); the second group with small pieces of crisps (5 mm^2^ of naturally flavored crisps Vico^®^, *n* = 15); and the third group received artificial cerebrospinal fluid (aCSF, Phymep, France) (*n* = 15). A fourth yoked-control group (yoked, *n* = 16) was submitted to the same protocol as morphine-rewarded animals, except that they could not trigger any injection. Instead the computer did so each time a paired self-administering animal reached the correct arm, so that the number of morphine injections (and thus the dose) received by yoked controls was equivalent but irrespective of their behavior or location in the maze, as previously described (35).

Small pieces (5 mm^2^) of naturally flavored crisps were chosen as food reward after pilot studies showing that motivation to learn the task was obtained with a very low level of deprivation (<5%). Therefore, the same level of deprivation was applied to all groups to ensure a comparable physiological state in all animals. Intracranial drug self-administration was used as a model of reinforcement learning similarly to intracranial self-stimulation (47). This model presented several advantages. Food or drugs were self-administered in the same conditions, avoiding manipulation during behavioral tests, thus allowing direct comparison of learning in drug and food-reinforced animals. We used morphine as a mean to activate pharmacologically VTA–DA neurons without altering directly function in all brain regions (35). The dose of morphine was selected on the basis of optimal learning performance established in dose–effect curves reported previously using the same task (48).

#### The Water-Maze Competition Task

The test used is an adaptation of the previously published water-maze competition task in the mouse (6, 13, 38). The training regimen is an important factor in the modulation of interactions between memory systems (49, 50). We used an acquisition protocol allowing a balanced expression of HPC and striatum-dependent learning (13). The last training session of the Y-maze learning task was followed by a 72 h-resting period after which the water-maze task started in a subgroup of mice [*n* = 33, composed of the following: morphine reward (*n* = 8); crisp reward (*n* = 8); aCSF (*n* = 9); and yoked morphine (*n* = 8)]. This delay allowed for a complete washout of morphine from the animal’s brain (51), thus avoiding any effect of residual morphine on brain function during the competition task. Briefly, the task is composed of two stages. During the acquisition phase (10 trials, ITI 10 min), animals start from a constant position and have to reach a submerged platform located by both a cue in its center and numerous extra-maze visual cues. The platform remained in a fixed position for the whole acquisition phase. On the following day, mice underwent the retention test (five trials, ITI 10 min). One platform remained in the spatial location learnt the day before, whereas a second, new platform marked by the cue used during acquisition was introduced and located in the opposite quadrant. The starting point was changed to be equidistant from both platforms.

#### Immunohistochemistry

Concurrently to the WM competition task, i.e., 72 h after completion of the Y-maze training, brains of another subgroup of mice [*n* = 30; composed of the following: morphine reward (*n* = 8); crisp reward (*n* = 7); aCSF (*n* = 7); and yoked morphine (*n* = 8)] were removed to assess changes in brain regional expression of pCREB as previously described (41). We used unbiased stereology in the following areas according to Paxinos and Franklin (52): subfields of the dorsal HPC (CA1, CA3), the DS, the shell part of the NAC, and prefrontal cortex (infralimbic and prelimbic parts merged) (PFC). Cell counts were expressed as mean number of pCREB positive nuclei per square millimeters. Under anesthesia, animals were perfused transcardially with a cold (4°C) solution of 4% paraformaldehyde in PB (0.1 M, pH 7.4). Brains were then removed and postfixed overnight in the same fixative at 4°C. Brains were then put in a saccharose solution (30% in Tris buffer 0.1 M, pH 7.4) over a night and were then frozen to make 50-μm coronal free-floating sections with a freezing microtome (Leica) to proceed the pCREB immunochemistry. All solutions contained the phosphatase inhibitor sodium fluoride (2.1 g/L). Sections were collected in Tris buffer (0.1 M). After elimination of endogenous peroxidase activity by H_2_O_2_ 30 min incubation and a preincubation step in saturation buffer (bovine serum albumin 1%, goat serum 3%, Triton X100 0.2%), sections were incubated for 48 h with rabbit anti-pCREB antibody (1:6,000 in saturation buffer, Millipore, Billerica, MA, USA). Subsequently, sections were incubated with biotinylated goat anti-rabbit antibody (1:2,000 in Tris buffer, Jackson Immunoresearch) and followed by an avidin-biotinylated horseradish peroxidase complex (Vectastain Elite Kit, Vector Laboratories, Burlingame, CA, USA). The peroxidase reaction end product was visualized in a Tris solution containing diaminobenzidine tetrahydrochloride (5%). Sections were mounted on gelatin-coated slides, air-dried, dehydrated, cover slipped with Eukitt and examined through light microscopy. The quantification of pCREB positive nuclei was carried out at 10× magnification, which yielded a field of view of 849 μm × 637 μm. At least six serial sections for each brain regions were digitized bilaterally and analyzed using a computerized image analysis system (Biocom, Visiolab 2000, V4.50). The number of nuclei was quantified blind to experimental conditions.

### Experiment II: Inhibition of PKA Activity within the DS

#### Surgery

An additional cohort of mice (*n* = 15) received a guide cannula 1.5 mm above the VTA and were implanted bilaterally with two guide cannulae (gauge 30) 1 mm above the mediolateral midline of the DS (from Bregma: AP: +0.5 mm, ML: ±1.9 mm, DV: −2.0 mm from skull surface), so that the stainless-steel injection cannulae (gauge 36) used for bilateral infusions projected to 1 mm below the tip of the guide-cannula.

#### Rp-8Br-cAMPs Infusions

The 8-bromoadenosine-3′,5′-cyclic monophosphorothioate, Rp-isomer (Rp-8Br-cAMPS; Enzo Life Science) is a lipophilic analog of Rp-cAMPS, a well-characterized membrane-permeable competitive inhibitor of cyclic AMP-dependent protein kinase (PKA), which discriminates between PKA and other cAMP receptors (53). On the basis of previous behavioral and CREB expression studies in C57BL/6 mice (35, 54), Rp-8Br-cAMPS was dissolved in aCSF to be delivered at the concentration of 0.4 nmol/0.5 μl per hemisphere. Bilateral infusions were performed before the last Y-maze session to avoid disruption of encoding during the water-maze task that was run 72 h after. Ten minutes before the last training session, mice were injected for 3 min in their home cage with either the Rp-8Br-cAMPS (*n* = 6) or aCSF (*n* = 6) into the DS, using a double infusion pump (Elite 11, Harvard^®^). Injectors remained connected for 2 min after the injection. Mice were then allowed to rest for 5 min.

### Statistical Analysis

#### Y-Maze

The mean number of correct responses and the mean choice latency per trial were analyzed using a two-way analysis of variance (ANOVA) (StatView 5.01 statistical software, Abacus Concept, Piscataway PA, USA) with “Reward” type as between-subjects factors and “Session” as a within-subjects repeated factor. Day-by-day between-groups comparisons for latencies and responses were performed using a one-way ANOVA with “Reward” as between subject factor. Significant main effects were further analyzed (*post hoc*) using Newman–Keuls *t*-tests. One sample *t*-tests were used to compare performance in the last training session against chance level (5/10 correct responses).

#### Water Maze

Analysis of the swim distance within the acquisition or retention phase was performed using a two-way ANOVA with “Reward” type as between-subjects factors and “Trial” as within-subjects repeated factor. Mean swim speed over all acquisition or retention trials was analyzed using a one-way ANOVA with “Reward” as between subject factor. For the water-maze retention test, the percentage of cue or place responses and the percentage of time spent in enlarged platform were compared across groups using unpaired Student’s *t*-test.

#### Immunochemistry

Immunostaining data were expressed as mean number of pCREB positive nucleus per square millimeters for each of both hemispheres. Six consecutive serial sections were examined bilaterally for all regions. We found no left–right difference; therefore, data were averaged to produce group mean ± SEM. One-way ANOVAs with “Reward” as between-group factor followed by *post hoc* Newman–Keuls *t*-tests were performed.

## Results

### No Differential Effect of Food vs Drug Rewards on Learning Performance in the Y-Maze Task

**Figure 3:**
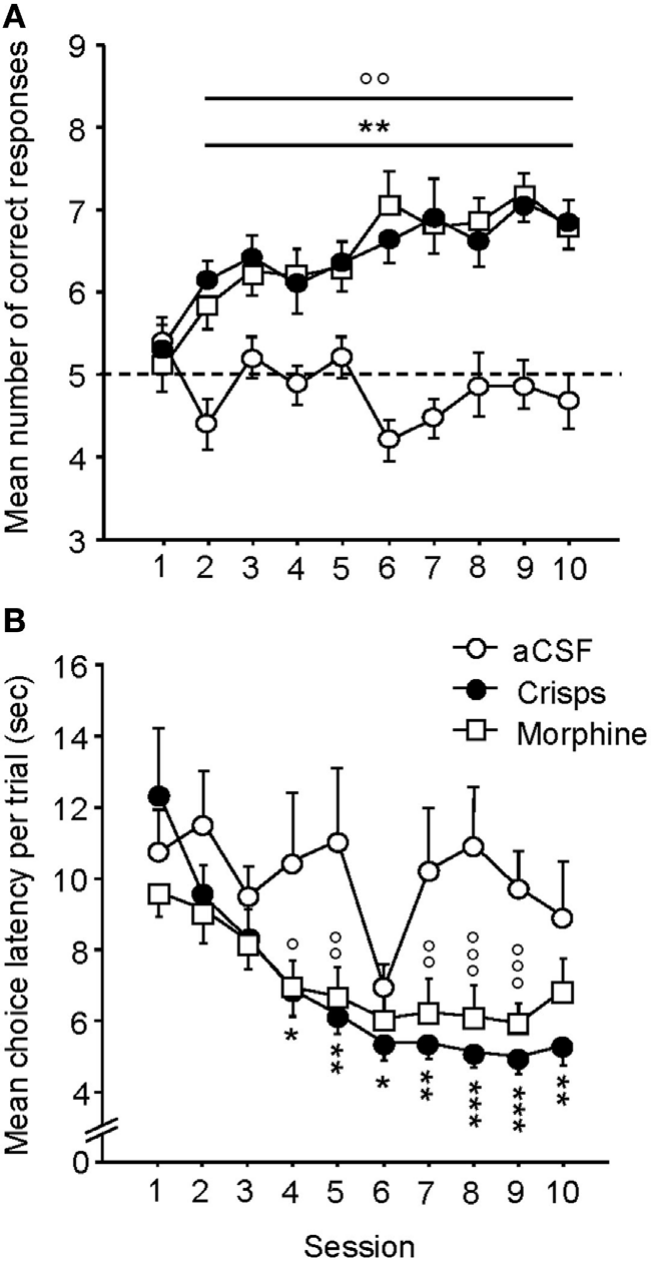
**Acquisition of the cue-guided Y-maze discrimination task in food (crisps) and drug (morphine) self-rewarded mice**. **(A)** Mean (±SEM) number of correct responses over 10 training sessions (10 trials/day). Both natural (“Crisps” group: *black dot*) and pharmacological (“Morphine” group: *white square*) rewards allowed the acquisition of this task as compared to artificial cerebrospinal fluid (aCSF) (*white dot*) injected group (vs Crisps group from day 2 to 10: **p < 0.01; vs Morphine group from day 2 to 10: ^°°^*p* < 0.01). **(B)** Analysis of mean (±SEM) latencies to complete a trial (in seconds) over the 10 training sessions. Both rewarded groups decrease their choice latency over trials and completed trials faster than aCSF group (vs Crisps group: *p < 0.05; **p < 0.01; ***p < 0.001; vs Morphine group: ^°^*p* < 0.05; ^°°^*p* < 0.01; ^°°°^*p* < 0.001).

As illustrated in Figure 3A, both crisp- and morphine-rewarded mice learned similarly the cue-guided Y-maze discrimination task. The number of correct responses for these two groups increased over sessions, whereas aCSF controls performed at chance level and did not improve across trials (two-way ANOVA: Reward effect: *F*_2,44_ = 46.90, *p* < 0.001; Session effect: *F*_9,396_ = 4.18, *p* < 0.001; Reward × Session interaction: *F*_18,396_ = 3.18, *p* < 0.001; *post hoc*: Crisps vs aCSF *p* < 0.001; Morphine vs aCSF, *p* < 0.001; Morphine vs Crisps, *p* > 0.05). Both Crisp- and Morphine-rewarded mice choose the reinforced arm significantly more than aCSF controls from day 2 to day 10 (all *p* < 0.05) and displayed very similar learning rates as evidenced by their overlapping learning curves. Analysis of the mean latency to complete trials (Figure 3B) revealed that this parameter significantly decreased over sessions in both morphine- and crisp-rewarded mice, but not in mice that received aCSF (Reward effect: *F*_2,44_ = 8.72, *p* < 0.001; Session effect: *F*_9,396_ = 8.38, *p* < 0.001; Reward × Session interaction: *F*_18,396_ = 2.23, *p* = 0.027; *post hoc*: Crisps vs aCSF, *p* < 0.01; Morphine vs aCSF, *p* < 0.01; Morphine vs Crisps, *p* > 0.05).

### Morphine Self-administration Elicits Long-lasting CREB Phosphorylation in the DS while Reducing pCREB Expression in the HPC

**Figure 4:**
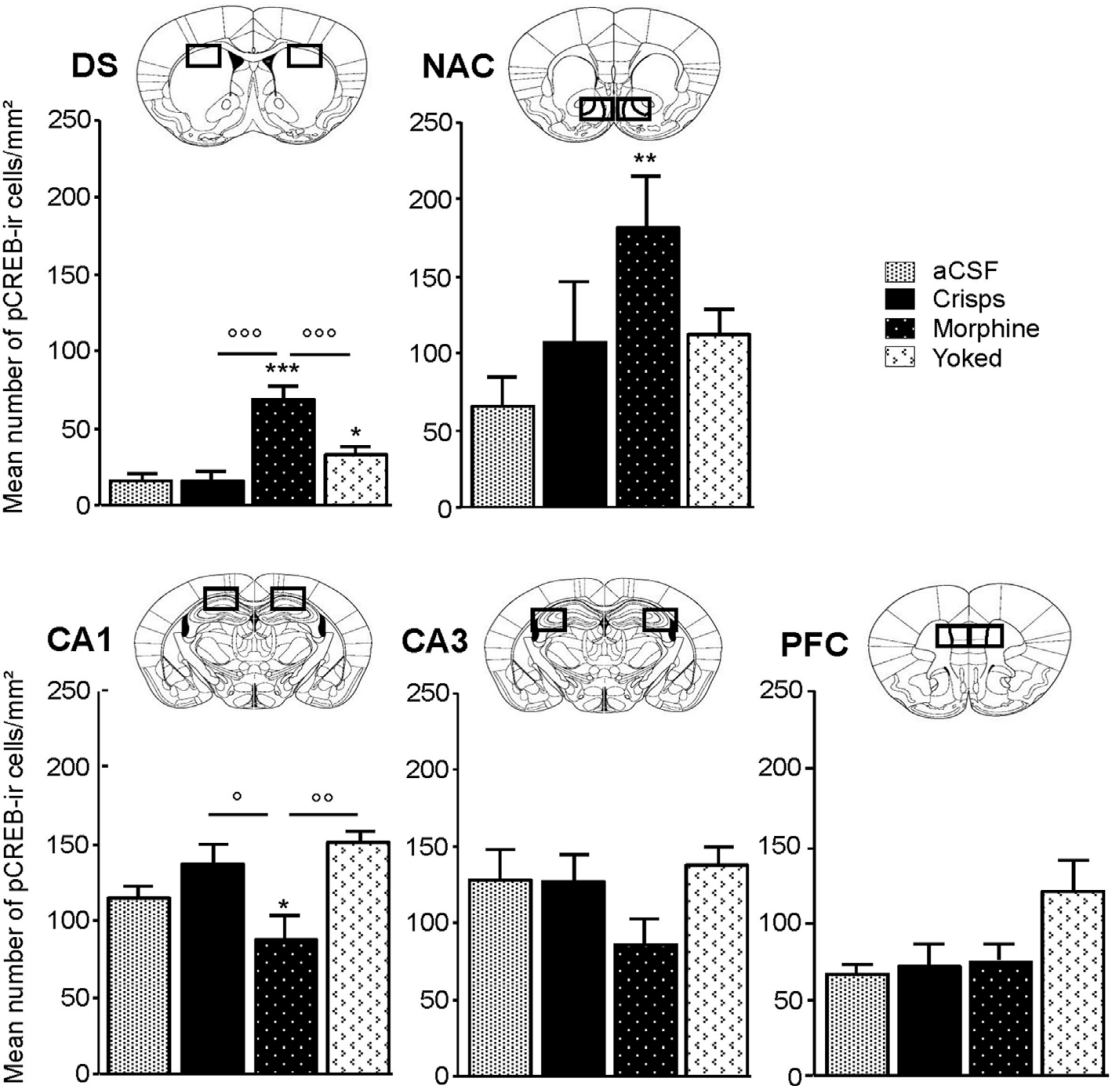
**Region-specific patterns of CREB phosphorylation 72 h after the last session of the Y-maze discrimination learning task**. Measures were expressed as mean (±SEM) number of pCREB immunoreactive cells (pCREB-ir) per square millimeters in the dorsal caudate putamen or striatum, dorsal striatum (DS), nucleus accumbens (NAC) shell, subfield CA1 of the dorsal hippocampus (HPC) (CA1), subfield CA3 of the dorsal HPC (CA3) and prefrontal cortex (PFC). Comparison with artificial cerebrospinal fluid (aCSF) control group: *p < 0.05; **p < 0.01; ***p < 0.001; other comparisons: ^°^*p* < 0.05; ^°°^*p* < 0.01; ^°°°^*p* < 0.001.

**Figure 5:**
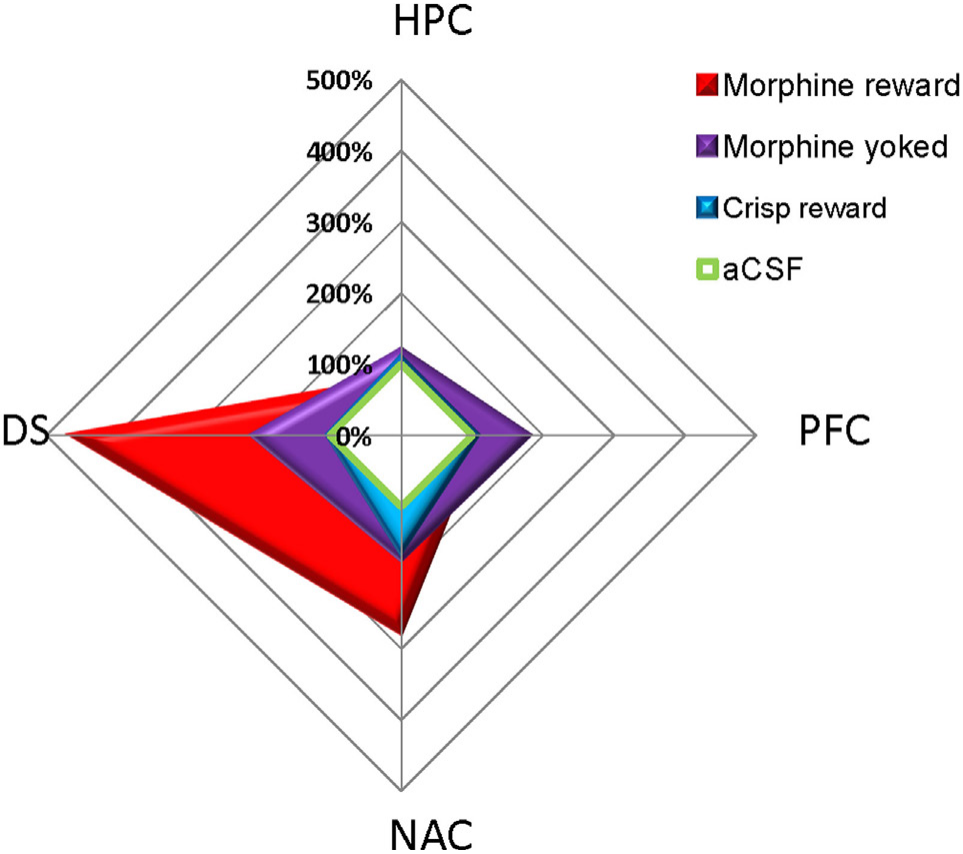
**Summary of pCREB immunostaining changes relative to artificial cerebrospinal fluid (aCSF) animals (100%) 72 h after the last session of the Y-maze discrimination learning task**. Previously drug (morphine) but not food (crisps)-rewarded animals exhibited a drastic and persistent increase in pCREB levels in the dorsal striatum (DS) and, although to a much lesser extent, in the nucleus accumbens (NAC) shell. In contrast, yoked controls exhibited an undifferentiated pattern of regional expression.

pCREB immunostaining was performed to reveal the brain regional activation state in animals of each group 72 h after the last Y-maze session. Expression levels are detailed in Figure 4. At this delay, previously food rewarded and aCSF controls exhibited similar pCREB levels in the analyzed structures. In contrast, morphine-exposed animals exhibited higher pCREB levels as compared to other groups in the DS, and this effect was significantly heightened when morphine was self-administrated as compared with yoked subjects (Reward effect: *F*_3,26_ = 26.70, *p* < 0.001; *post hoc*: Morphine vs aCSF, *p* < 0.001; Morphine vs Crisps, *p* < 0.001; Morphine vs Yoked, *p* < 0.001; Yoked vs aCSF, *p* = 0.04; Yoked vs Crisps, *p* = 0.03). Statistical analysis also yielded an elevated level of pCREB in the NAC of morphine self-administering mice (Reward effect: *F*_3,26_ = 3.19, *p* = 0.039; *post hoc*: Morphine vs aCSF, *p* = 0.006; Morphine vs Crisps, *p* = 0.056; Morphine vs Yoked, *p* = 0.071). In contrast, pCREB expression in the dorsal CA1 of the HPC was significantly reduced in mice with a history of morphine self-administration (Reward effect: *F*_3,26_ = 4.21, *p* = 0.014; *post hoc*: Morphine vs Crisp, *p* = 0.02; Morphine vs Yoked, *p* = 0.002; Morphine vs aCSF, *p* > 0.05). A similar, although non-significant tendency was observed also in the CA3 (Reward effect: *F*_3,26_ = 1.30 ns). In the PFC, pCREB levels were slightly elevated in Yoked subjects but this effect did not reach significance (Reward effect: *F*_3,26_ = 2.83 ns). Figure 5 summarizes region-dependent relative changes and points out to a drastic increase in the DS, but a decrease in the dorsal HPC (CA1–CA3).

### History of Morphine Self-administration Promotes Cue-Guided Learning Strategy

**Figure 6:**
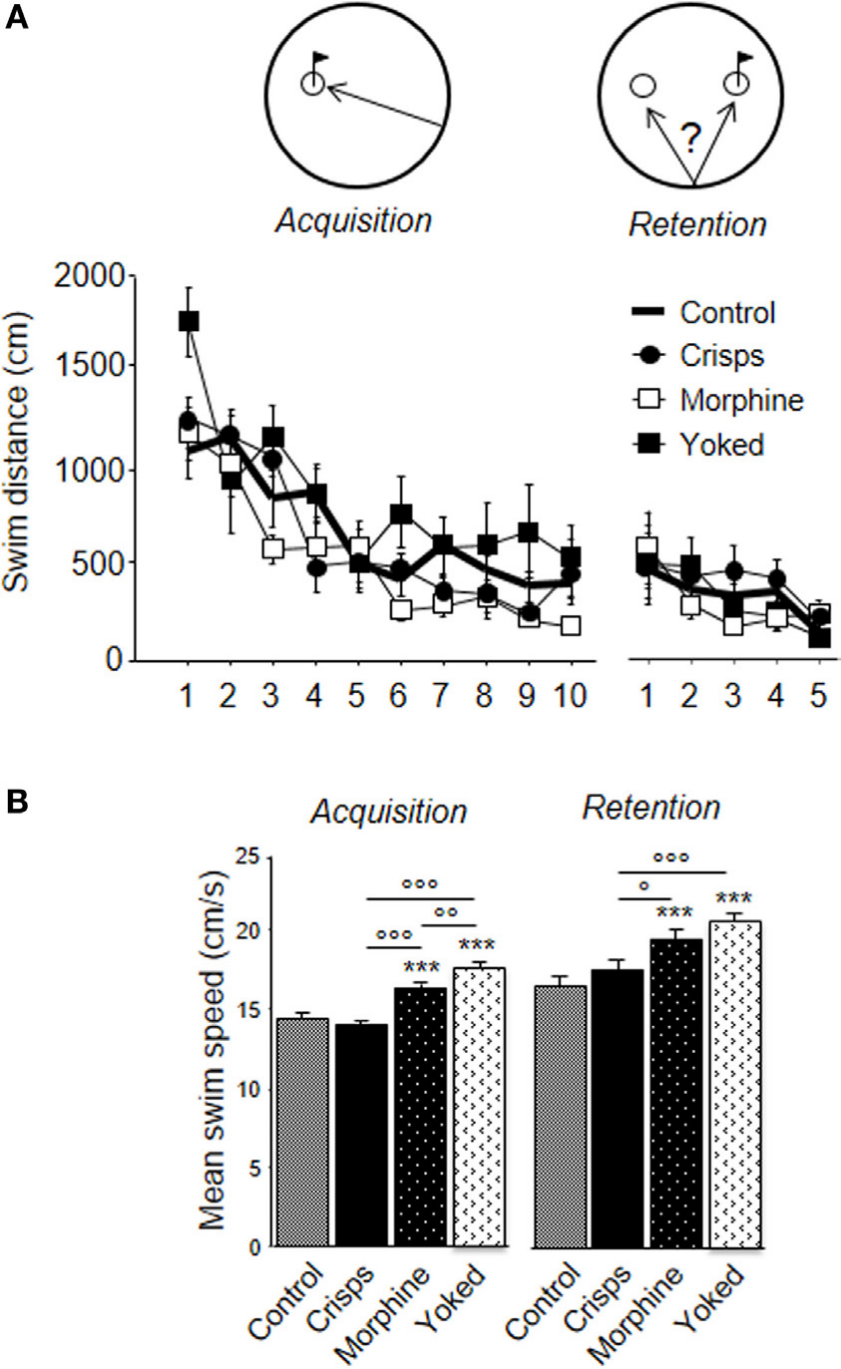
**Learning parameters in the water-maze competition task**. **(A)** All animals learnt to retrieve more precisely the hidden platform during the acquisition phase. During the retention phase, animals reached a plateau and did not further decrease their latency to escape. **(B)** Analysis of swim speed revealed that mice previously injected with morphine swam faster during both phase of the water-maze task than Crisps (^°^*p* < 0.05, ^°°^*p* < 0.01, ^°°°^*p* < 0.001) and artificial cerebrospinal fluid group (***p < 0.001).

As shown on Figure 6A, all animals learned to find the platform efficiently over trials. However, the previously morphine-rewarded group displayed better learning performance than aCSF-injected animals, whereas subjects having experienced non-contingent morphine administrations (yoked controls) had to swim more than any other groups (ANOVA Reward effect: *F*_3,29_ = 6.71, *p* = 0.001; Trial effect: *F*_9,261_ = 24.35, *p* < 0.001; *post hoc*: Morphine vs aCSF, *p* = 0.03; Yoked vs aCSF, *p* = 0.02; Yoked vs Morphine, *p* = 0.001; Yoked vs aCSF, *p* = 0.009; Crisps vs aCSF, n.s.; Crisps vs Morphine, n.s.). These differences were abolished during the competition task. Analysis of the mean swim speed over acquisition trials pointed to group differences (Reward effect: *F*_3,326_ = 26.57, *p* < 0.001): previously drug-rewarded mice swam faster than food-rewarded subjects (all *p* < 0.001) and aCSF controls (all *p* < 0.001) (Figure 6B). These differences were observed also in the retention test (Reward effect: *F*_3,161_ = 11.26, *p* < 0.001; Yoked vs aCSF; Yoked vs Crisps and Morphine vs aCSF, *p* < 0.001; Morphine vs Crisps *p* = 0.02).

**Figure 7:**
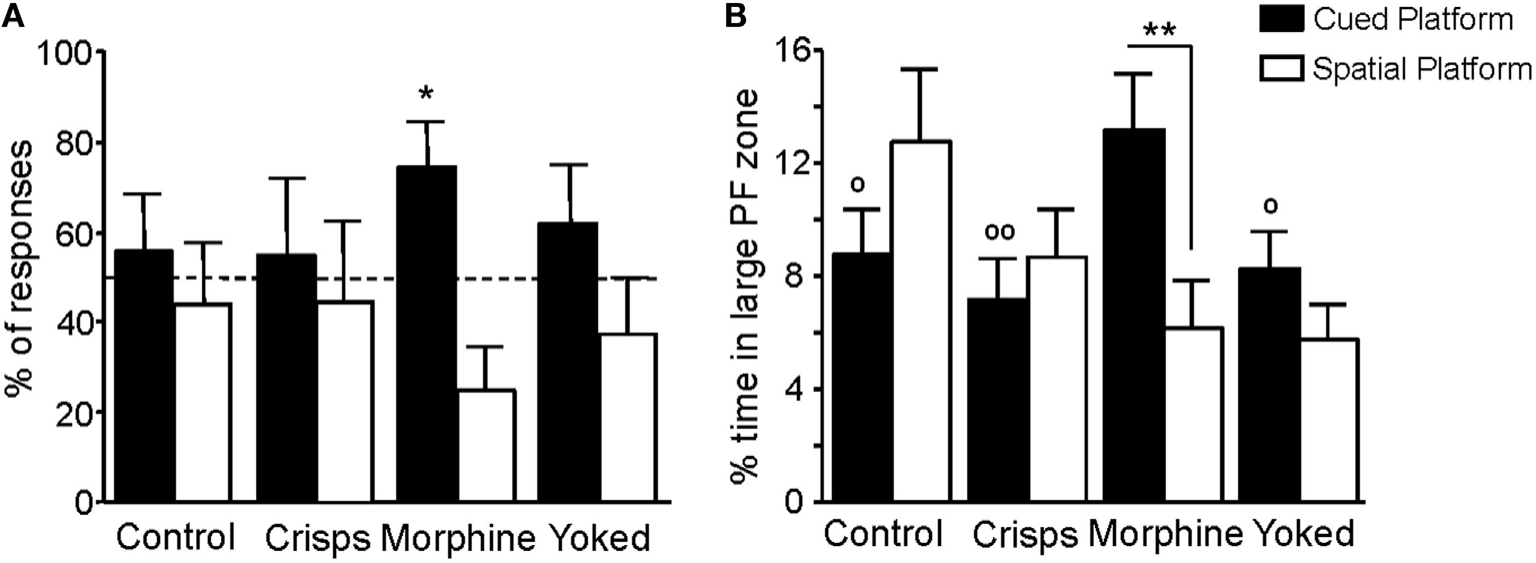
**Navigation strategies used during the retention phase**. **(A)** Previously morphine-self-administering mice exhibited a strong preference for the cued platform as expressed by the percentage (±SEM) of responses made within the five retention trials (*p < 0.05). **(B)** Analysis of percentage of time spent in each large platform zone showed that artificial cerebrospinal fluid, Crisps, and Yoked groups swam the same amount of time in both part of the maze whereas morphine animals swam more in the cued large platform zone (within group comparison: **p < 0.01; vs Morphine: ^°°^*p* < 0.01, ^°^*p* < 0.05).

Spatial vs cue-oriented responses during the retention test are shown in Figure 7A. Behavior of previously drug self-administering mice was dominated by the single cue, whereas behavior of food-rewarded, yoked, and aCSF control animals was equally influenced by spatial information and the cue (*t-*test vs chance level of 50%: Morphine *t* = 2.75, *p* = 0.02; aCSF, Crisps, Yoked all *p* > 0.20). Animals that had experienced morphine self-administration earlier on spent more time in the enlarged cued-platform zone than all the other groups (Reward effect: *F*_3,161_ = 2.66, *p* < 0.05; *post hoc* tests: Morphine vs aCSF, *p* < 0.05; Morphine vs Crisps, *p* < 0.01; Morphine vs Yoked, *p* < 0.05). Moreover, morphine self-administered animals swam more in the enlarged cued-platform zone than in the spatial one during retention trials (unpaired *t-*test: Morphine, *p* = 0.008; Crisps, Yoked, and aCSF all *p* > 0.05; Figure 7B).

### Inhibition of PKA/CREB Pathway in the DS Abolishes the Bias toward Cue-Oriented Learning

**Figure 8:**
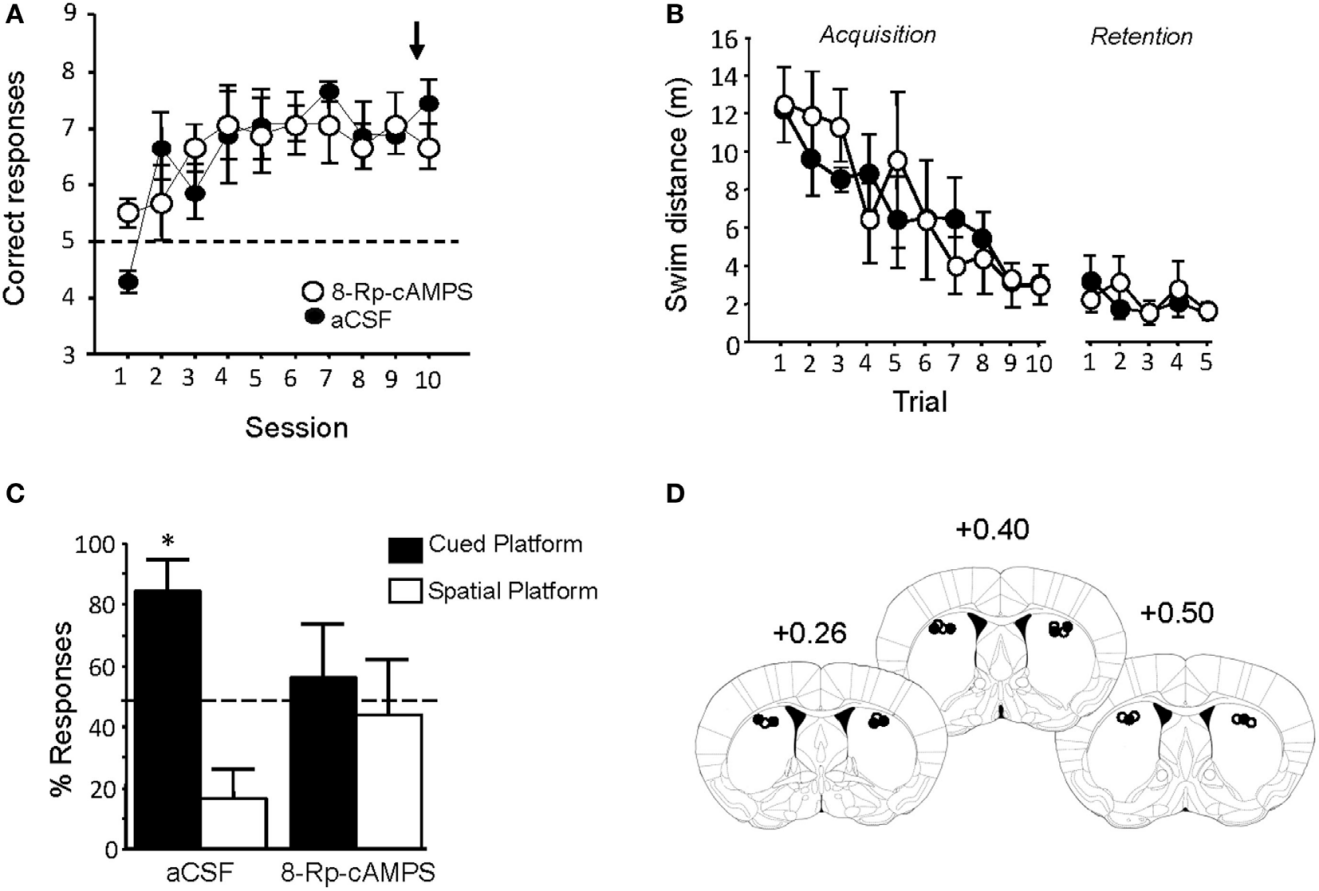
**Rp-8Br-cAMPS infusions into the dorsal striatum reverse preferential use of a cue-guided strategy**. **(A)** PKA inhibition before the last Y-maze session did not impair the previously learned behavior. **(B)** Rp-8Br-cAMPS infusions had no effect on swim distance required to locate the hidden platform during both acquisition and retention phase of the WM competition task. **(C)** Morphine animals infused with artificial cerebrospinal fluid (aCSF) preferred the cued platform (*p < 0.05). Rp-8Br-cAMPS-treated mice exhibit no preference for a particular learning strategy over the five retention trials (*p* > 0.05). **(D)** *Lef*t: schematic representation of injection sites (*white dot*: aCSF injection sites; *black dot*: Rp-8Br-cAMPS injection site). *Right*: histological report of injection sites for all pretreated animals (numbers refer to AP stereotaxic coordinates relative to Bregma).

Pre-injection of Rp-8Br-cAMPS had no effect on performance during the last Y-maze acquisition session (Figure 8A). Treated animals were tested in the water-maze competition task 72 h later. Rp-cAMPS or aCSF injections into the DLS did not alter swim distances to the platform during either the acquisition or retention phase of the water-maze task (Figure 8B). Rp-8Br-cAMPS pretreatment, however, completely abolished the preferential use of the cue-guided learning strategy that was observed in aCSF treated mice. As evidenced by the percentage of responses over the five retention trials summarized in Figure 8C, Rp-8Br-cAMPS-treated animals displayed as many spatial as cue-oriented responses (*t*-test against theoretical 50% chance level: *p* > 0.05), whereas subjects receiving the vehicle persisted in choosing the cued platform over the spatial platform (*t-*test against chance level: *t* = 3.47, *p* = 0.02). Histological control of all pretreated animals showed that injection sites were located mainly in the DLS (Figure 8D), as can be estimated from the study of Yin and Knowlton (55).

## Discussion

We previously reported that drug-reinforced animals are selectively impaired in the acquisition of a spatial discrimination task, but not in the cued version of the same task (35). This finding suggests that drug rewards may induce a shift toward cue-oriented behavior and striatum-dependent forms of learning. In the present study, we challenged this view by assessing the selection of spatial vs cue-oriented learning strategies in a water-maze competition task (13). We compared mice having experienced a Y-maze discrimination task rewarded with either food, non-contingent or self-administered morphine. We now show that animals with a history of drug self-administration rely almost exclusively on a cue-guided strategy to reach the platform. In contrast, animals having received passively the same amount of morphine as well as food-rewarded subjects, retained a flexible use of spatial and cued strategies. Along with their cue-dependent behavior, animals with a history of morphine self-administration displayed a persistent increase in pCREB within the DS and the NAC, but a decrease in the dorsal CA1. This expression pattern was bilateral, thus ruling out any possibility that unilateral activation of these brain regions may underlie cognitive inability. Such an inverse relationship between striatal and hippocampal pCREB expression as demonstrated by present behavioral, CREB-imaging, and pharmacological data fits well with the view that a functional antagonism between HPC and DS takes place during learning. Consistently, decreasing HPC function or enhancing DS processing using pharmacological or genetic manipulation of pCREB levels induces a predominant use of striatum-dependent learning in navigational tasks (12, 38, 39, 56). Humans using response strategies in navigational tasks exhibit increased fMRI activity and gray matter in the DS (57, 58).

The habit-forming effects of drugs of abuse are well documented (3, 59). Repeated systemic or intra-VTA administration of amphetamine or morphine induces an increase in locomotor activity and repetitive, stereotyped behaviors (60–62). This behavioral sensitization can disrupt action–outcome (A–O) learning, and repeated preexposure to a psychostimulant promotes habitual responding in a DA-D1 receptor-dependent manner (63, 64). We show here that VTA morphine reward not only promotes S–R learning but it also increases the bias toward subsequent striatum-dependent learning. This is consistent with the view that repeated cued drug self-administration facilitates the use of striatum-dependent learning strategies (65). This cue attractiveness could be related to a sign-tracking profile as recently defined in rats (66). Sign-tracking refers to individuals more likely to approach cues in a novel environment, whereas goal trackers will try to locate directly the reward (food tray). Interestingly, sign trackers exhibit phasic DA signals shifting from the unconditional stimulus (US food) to the conditional stimulus (CS cue), whereas goal trackers maintain an elevated DA response to the CS and US. Rats selectively bred for high reactivity to a novel environment show a sign-tracking response and an increased propensity to self-administer cocaine, suggesting that they could represent an animal model of addiction vulnerability (67). Identification of common neural features of sign-tracking (rat) and cue attractiveness (mouse) is an interesting prospect for future addiction research.

There is ample evidence that cue-dependent control of behavior in drug addiction relies on neuroadaptations occurring in the PKA/pCREB signaling pathway within cortico-limbic-striatal and amygdala circuits (1, 68–70). Chronic drug use led to an aberrant over-learning of drug-related cues, and craving or relapse can be induced by presenting such cues (71–73). Here, we provide evidence that morphine self-administration upregulate CREB activity within the DS, facilitating the recruitment of a learning strategy depending on cues. Concurrently, pCREB level was reduced in dorsal CA1 of the HPC, a region involved in flexible, spatial learning. Reward-dependent increase in striatal DA facilitates LTP at the level of medium spiny neurons of the direct pathway (74), and this form of LTP depends on D_1_-DA receptors or co-activation of D_1_/NMDA receptors (75, 76). Chronic drug-induced modulation of DA D_1_/D_2_ receptor ratio in the DS leads to an increased excitability of this brain region in humans (77). Together, these data strongly suggest that drug-reinforced learning resulted in hyperactivity of the DS. Consistently, we show that blocking striatal PKA activity with Rp-8Br-cAMPS restored a balanced expression of cued and spatial navigation strategies. PKA is the main kinase involved in CREB phosphorylation through DA D_1_ signaling (78–80). PKA activity maintains cue-dependent control of behavior through a DA/glutamate signaling cascade (68). Importantly, CREB may be phosphorylated also *via* the extracellular signal-regulated kinase pathway, its recruitment depending mainly on glutamatergic inputs (81–83). The efficiency of Rp-8Br-cAMPs in restoring spatial learning could reflect either a predominant role of the DA-dependent striatal PKA, or an alteration of coincident DA-glutamate signaling. In any case, it is consistent with a role of DS DA in navigational tasks (55, 84), the inhibiting effects of DS electrical stimulation on the HPC (85), and the improving effect of DS lesions on spatial learning (12).

Since we previously demonstrated that Rp-8Br-cAMPS did not blocked CREB activity in the adjacent ventral striatum, it is unlikely that this inhibitor had to reach distant, extra-striatal regions to exert its effect (35). This view is also supported by the observation that transgenic mice expressing a dominant-negative mutant of CREB show specific impairments in both CREB activity in the DS and cued learning (12). However, at least three subregions have been described within the DS itself based on functional data: the anterior dorsomedial, the posterior dorsomedial, and the DLS (37, 55, 86–90). One limitation of our PKA/CREB inhibition study is that Rp-8Br-cAMPS injections targeted the midline of the DS; therefore, it is not possible to attribute its effects selectively to one of these subregions. Yet, histological control points out to the DLS, thus present restorative effects of PKA inhibition on place learning are consistent with the lateral/medial dissociation of the DS, respectively, associated with habitual/A–O responses in instrumental and drug-maintained behaviors, or response/place learning (37, 55, 86–90). Finally, since food-trained mice exhibited neither persistent CREB activity nor learning bias in the WM competition task, they were not tested for Rp-8Br-cAMPS, leaving open the question of its action in non-biased animal. We and others have reported that the effects of PKA inhibitors on memory typically depend on the region that is targeted: intra-HPC administration blocks spatial memory, whereas intra-DS and intra-PFC infusions disrupt striatum-dependent learning and cued-induced relapse (35, 91–93).

One intriguing observation of the present study is that yoked morphine did not have the same cognitive impact than self-administered morphine. During the Y-maze task, all mice were trained on a cued protocol, raising the possibility that a morphine-training interaction might explain subsequent preference for the cued learning strategy. The absence of preferential cued learning (and DS-CREB hyperactivity) in the yoked-control group, in which each subject received non-contingently the same amount of morphine as self-administering animals, demonstrates that this interaction is not sufficient to elicit this learning bias. Instead, it suggests that response contingency is involved in this form of neuroplasticity. Profound differences between self-administered and yoked cocaine rats have been reported in electrically evoked [(3)H] DA release (94). Self-administering animals exhibit sensitized DA release in the NAC, DS, and medial prefrontal cortex up to 3 weeks after cessation of cocaine self-administration, whereas terminal DA release is sensitized only in the NAC core in yoked subjects (94). Although the response contingency is clearly necessary, it is not sufficient to elicit such a cognitive bias, as it was not observed in food-rewarded animals. Our results suggest that reward value may be another critical component required for this long-lasting behavioral/cellular plasticity. The strong morphine-induced CREB activity observed in the NAC argues in favor of this hypothesis. Indeed, there is evidence that the reinforcer value plays a role in the facilitation of S–R learning (64).

There are striking similarities in the impact of emotional events on learning processes, whether their valence is positive (reward) or negative (stress). Both stress and drugs promote habit learning (15–19). Mechanisms underlying this effect remain to be fully understood, yet it has been proposed that drugs favor S–R association by impairing retrieval or utilization of outcomes (3). A growing body of evidence suggests that in humans, chronic consumption of drugs of abuse impairs HPC- and PFC-dependent learning tasks (95, 96), whereas habit learning is mostly spared or even enhanced by drug consumption (30, 97, 98). Accordingly, our results further reveal that morphine self-administration leads to a functional imbalance between the HPC and DS, prompting the use of the striatal-dependent habit learning system. Future work should aim at detecting a similar hippocampostriatal unbalance in human abstinent drug users, using functional or structural brain imaging. Enduring states of differential excitability could represent a form of disconnection syndrome contributing to the maintenance of addictive behaviors. Interestingly, young adults expressing a response learning strategy in a virtual navigational task use more drugs than spatial learners (99). These data raise a critical question awaiting to be specifically addressed by future research: could emotional events such as rewards, stressors, or even prenatal stress promote the habit system early on in life (100)? A corollary issue with tremendous therapeutic interest is whether or not pharmacological treatments or cognitive therapies aiming at restoring the HPC activity could maintain protracted abstinence or prevent relapse.

In conclusion, we provide behavioral, pharmacological, and cellular evidence suggesting that morphine reward elicits a cognitive bias toward the use of cue-guided learning strategies, an effect specifically observed in animals receiving contingent drug injections (self-administration). This cognitive bias relies on the persistent upregulation of learning-induced CREB phosphorylation in the DS and could be reversed by locally inhibiting the PKA/CREB signaling pathway. We suggest that such drug-induced biases are likely to play a critical, yet overlooked role in addictive behaviors, as they could counteract pharmacological treatments of addiction. This calls for further exploration of neural mechanisms involved in drug-induced cognitive biases toward cue-sensitive forms of learning.

## Author Contributions

MB, J-LG, VB, LS, and VD contributed to the writing of the manuscript. MB and MH performed experiments. MB, J-LG, and VD designed experiments.

## Conflict of Interest Statement

The authors declare that the research was conducted in the absence of any commercial or financial relationships that could be construed as a potential conflict of interest.
